# The detection rate and influencing factors of high-risk groups of cardiovascular disease in Anhui, China: A cross-sectional study of 99,821 residents

**DOI:** 10.3389/fpubh.2022.921038

**Published:** 2022-08-25

**Authors:** Xiu-Ya Xing, Zhi-Xin Wang, Ya-Wen Cao, Xin-Yi Wang, Luan Zhang, Ye-Ji Chen, Hua-Dong Wang, Jing-Qiao Xu, Mi-Xue Niu, Zhi-Rong Liu, Sha-Sha Tao

**Affiliations:** ^1^Anhui Provincial Center for Disease Control and Prevention, Department of Chronic Non-communicable Disease Prevention and Control, Hefei, China; ^2^Department of Rheumatology and Immunology, The First Affiliated Hospital of Anhui Medical University, Hefei, China; ^3^Major of Preventive Medicine, School of Public Health, Anhui Medical University, Hefei, China; ^4^Department of Radiation Oncology, The First Affiliated Hospital of Anhui Medical University, Hefei, China; ^5^First Clinical Medical College, Anhui Medical University, Hefei, China; ^6^Department of Epidemiology and Biostatistics, School of Public Health, Anhui Medical University, Hefei, China

**Keywords:** cardiovascular disease, high-risk groups, epidemiological characteristics, influencing factors, health education and promotion

## Abstract

**Objective:**

To investigate the detection rate and influencing factors of high-risk population of cardiovascular disease in Anhui province.

**Methods:**

From March 2017 to August 2019, the residents aged 35–75 years old were selected using the multi-stage stratified cluster sampling method in 8 counties and districts of Anhui Province, and questionnaire survey, anthropometric measurement, and collection of biological samples were carried out among them.

**Results:**

A total of 99,821 residents in Anhui Province were finally investigated, and among them 21,426 residents were detected to be high-risk groups of cardiovascular disease. The detection rate of high-risk groups was 21.46%. According to the high-risk types, the high-risk groups can be clustered. 74.57% of them had only one high-risk type, 22.57% of them had two high-risk types, and 2.86% had three or more high-risk types. The results of Generalized Linear Mixed Model (GLMM) showed that male, age ≥45 years old, not married, occupation as a farmer, annual family income <25,000 yuan, drinking, overweight and obesity, pre-central obesity and central obesity, snoring, feeling fatigued, sleepiness, and self-reported history of diabetes were more likely to be risk factors of cardiovascular disease (all *P* value < 0.05).

**Conclusion:**

The detection rate of high-risk groups of cardiovascular disease in Anhui Province is relatively high. Individualized intervention measures as well as comprehensive prevention and control strategies should be adopted focusing on the distribution characteristics of risk factors of high-risk groups.

## Introduction

Cardiovascular disease (CVD) is one of the most important public health issues in the world, which is the leading global cause of mortality, being responsible for 46% of non-communicable disease deaths (1). In China, the CVD mortality ranks first among all causes, accounting for more than 40% of resident disease deaths and the morbidity and mortality of CVD are still on the rise (2–4). China is also one of the countries with the heaviest CVD burden (5). In recent decades, CVD has been a serious health problem in China and the situation has not been alleviated (6). As we all know, people at high risk of CVD are very susceptible to CVD. Increasing evidences showed that early identification of high-risk groups and reasonable intervention and management for their corresponding risk factors can reduce the occurrence of cardiovascular events and premature the death caused by them (7). CVD is also the first cause of deaths in Anhui Province, and the crude mortality rate is still on the rise from 2013 to 2018 (8). However, there are few large-scale epidemiological studies on the investigation of high-risk groups of CVD in Anhui Province. The morbidity and mortality of CVD also vary among provinces or regions due to differences in geographical environment and dietary habits (9). This study organized the early screening of high-risk groups of CVD among community residents in Anhui Province from 2017 to 2019, and analyzed the current situation and related risk factors of high-risk groups, so as to detect high-risk groups in time, take corresponding intervention measures to reduce the occurrence of cardiovascular events in high-risk groups, and provide a basis for the prevention and control of CVD.

## Objects and methods

### Objects

**Figure 1:**
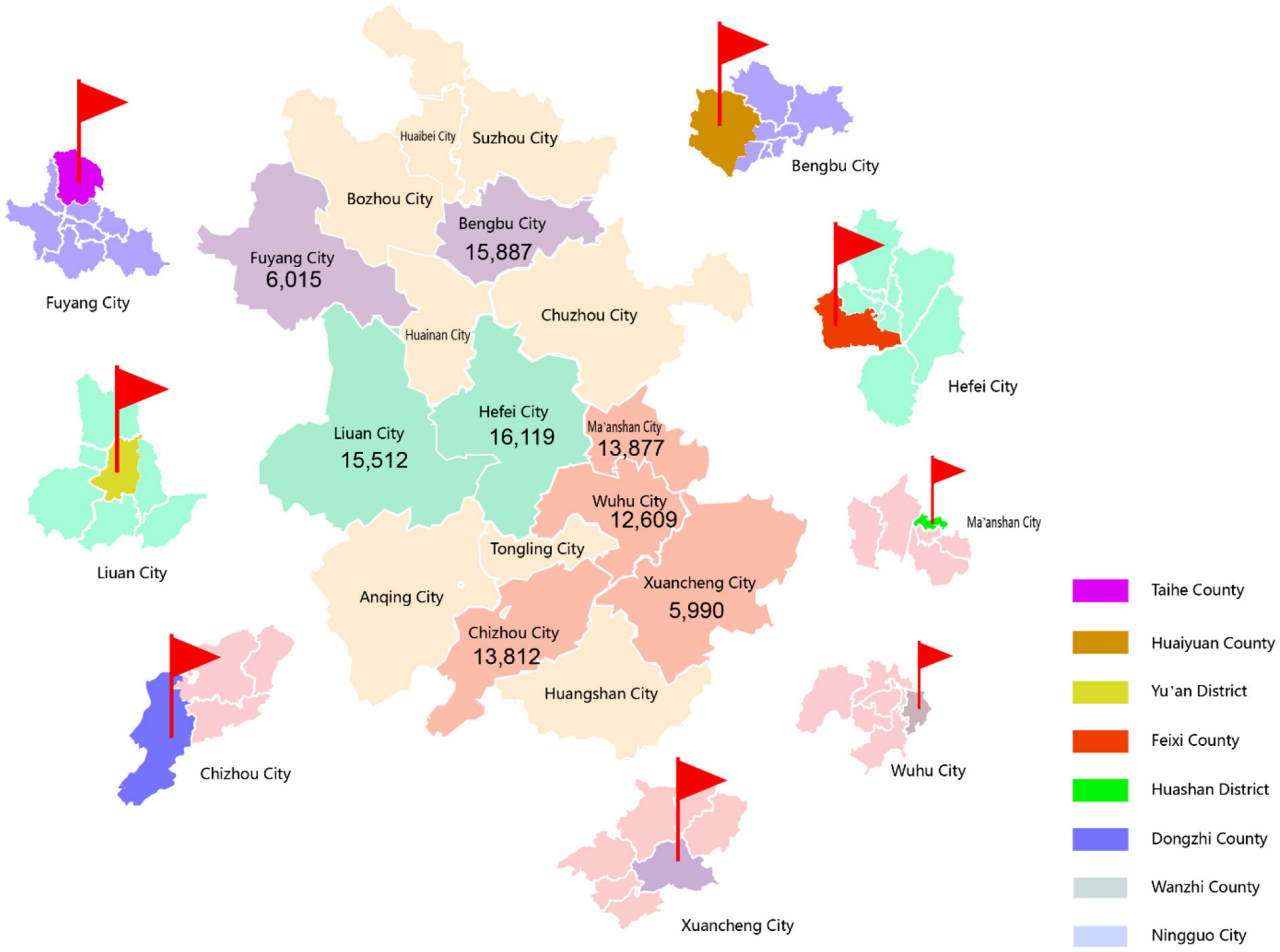
Eight counties/districts participating in the study.

When selecting samples, our investigation employed a convenience sampling approach with a four-level quality control (10). In addition, factors such as geographic location, economy, urban and rural resident population ratio, quality of disease and death registration, and local capacity to support programs were also considered. Ultimately, 8 out of 104 counties/districts in Anhui Province were selected between March 2017 and August 2019 (Figure 1). Among these 8 counties/districts, 4 counties/districts are in southern Anhui (Dongzhi County, Huashan District, Wanzhi County, Ningguo City), 2 counties/districts are in central Anhui (Feixi County, Yu'an District), and 2 counties/districts are in northern Anhui (Taihe County, Huaiyuan County). Among them, 2 counties/districts are urban areas (referring to prefecture-level cities) and six are rural areas. Secondly, we selected 2 to 8 townships or sub-districts from each county/district according to the size and the stability of local residents. Thirdly, the permanent residents aged 35–75 years old in these counties/districts were investigated. Written informed consent was obtained from all participants on entry into the project based on the principle of voluntary.

### Methods

Face-to-face investigations were conducted in a centralized post-appointment manner using an electronic data collection system. The main process and content of the primary screening included object identification, informed consent, information registration (collecting basic information such as name, gender, age, education level, occupation, marital status and so on), blood pressure, height, weight, waist circumference measurement, biological sample collection and biochemical indicator detection (detection of blood sugar and lipids and so on), questionnaire survey (collect information on smoking, drinking, hypertension, diabetes and so on). Cardiovascular risk calculation is based on WHO/ISH Cardiovascular Risk Prediction Charts for the Western Pacific Region B (11).

Since any personal identifiable information was not interviewed in this study, participants were informed that their participation was totally voluntarily and they can withdraw from the research during the investigation process without providing any reason, and it had no any adverse effects on the study subjects, thus only verbal informed consent was obtained from the research subjects prior to study commencement. All procedures were undertaken following the ethical standards of the Helsinki Declaration. Verbal informed consents were recorded and the study protocol was approved by The Committee on Medical Ethics of The First Affiliated Hospital of Anhui Medical University.

### Indicators and definitions

According to the criteria of high-risk subjects of CVD (12–14), since some survey objects can meet two or more high-risk types at the same time, there is overlap among objects of the four high-risk types, and high-risk subjects can be defined as one, two or more of the following four types at the same time: (1) CVD history: at least one disease history of myocardial infarction (MI), percutaneous coronary intervention (PCI), coronary artery bypass grafting (CABG), and stroke (ischemic or hemorrhagic); (2) hypertension: systolic blood pressure (SBP) ≥160 mmHg or diastolic blood pressure (DBP) ≥100 mmHg; (3)dyslipidemia: low-density lipoprotein cholesterol (LDL-C) ≥160 mg/dL (4.14 mmol/L) or high-density lipoprotein cholesterol (HDL-C) <30 mg/dL (0.78 mmol/L); and (4) high risk: using the information of age, gender, SBP and total cholesterol, smoking status and diabetes or not, the risk of CVD can be determined by a predetermined algorithm derived from the WHO/ISH Cardiovascular Risk Prediction Charts for the Western Pacific Region B (11). Individuals with a 10-years risk of CVD ≥20% are defined as high-risk subjects (10).

In this study, some survey objects can meet two or more high-risk types at the same time, so there is overlap among objects of the four high-risk types. The body mass index (BMI) (15) was used to determine overweight and obesity, and the standard adopted the National Health and Family Planning Commission of China (NHFPC) for Chinese adults, among which low weight: BMI <18.5 kg/m^2^, normal weight: 18.5 kg/m^2^ ≤ BMI <24.0 kg/m^2^, overweight: 24.0 kg/m^2^ ≤ BMI <28.0 kg/m^2^, obesity: BMI ≥ 28.0 kg/m^2^ (10). Using waist circumference to judge central obesity, waist circumference (WC) can be classified as normal (men: waist circumference <85 cm, women: waist circumference <80 cm), pre-central obesity (men: 85 cm ≤ waist circumference <90 cm, women: 80 cm ≤ waist circumference <85 cm), central obesity (men: waist circumference ≥90cm, women: waist circumference ≥85cm). The BMI categories of WHO for adults were as follows, low weight: BMI <18.5 kg/m^2^, normal weight: 18.5 kg/m^2^ ≤ BMI <24.9 kg/m^2^, overweight: 25.0 kg/m^2^ ≤ BMI <29.9 kg/m^2^ and obesity: BMI ≥ 30.0 kg/m^2^. Internationally, WC values >102 cm for men and >88 cm for women are generally classified as abdominal obesity (16). Person that self-reported current smoking and those who had smoked at least 1 day within the past 30 days was defined as smoker. Self-reported drinking alcohol within the past year when surveying was defined as drinker. A person with an affirmative answer of “Over the past 30 days, do you often feel tired, fatigued and sleepy?” would be classified as the person with fatigued and tired (10).

### Quality control

The procedures and the methods of investigation were uniform and strictly controlled. The investigators were all doctors or nurses, and they must undergo uniform training and pass the assessment before they participated in this survey. Moreover, we established a four-levels quality control system of province, city, county (district) and individuals in our survey. Quality control teams were set up at each level and professional quality supervisors were trained for quality control. Electronic questionnaires were used in our survey, so the data can be uploaded and updated in a timely manner.

### Statistical analysis

Socio-demographic characteristics were described using frequencies for categorical variables, mean and standard deviation (SD) for continuous variables. The χ^2^ test was used to compare the rate of residents at high risk of CVD in populations with different characteristics. Statistical analysis above all were performed using SPSS 23.0 software. Furthermore, since the sample in this study has a multi-level structure (urban vs. rural, 3 regions of Anhui province), Generalized Linear Mixed Model (GLMM) was used to further analyze the relationship between potential influencing factors of the high-risk groups of CVD, which were found in univariate analysis (*P* < 0.20), and the high-risk groups of CVD. In GLMM analysis, the two factors, “Urban or rural” and “Region” were analyzed as random intercepts. GLMM analysis were carried out with R 4.1.1 statistical analysis software. The significance tests are two-sided, with a *P* value ≤ 0.05 considered statistically significant.

## Results

### Socio-demographic characteristics of participants

In this study, a total of 99,821 residents in 8 counties (districts) were surveyed. There were 41,152 males (41.23%) and 58,669 females (58.77%), aged between 35 and 75 years, with an average age of (57.72 ± 9.91) years. The majority educational level of the residents was illiterate/semi-literate (47.80%). Among the residents, 66.19% of them were farmers, 91.74% of them were married, 40.27% of them had annual family income > 25,000 yuan, 70.56% of them lived in rural areas, 31.69% of them lived in middle Anhui, 46.37% of them lived in southern Anhui, 20.54% of them were smokers, 16.79% of them were drinkers, 53.70% of them were overweight and obese, and 38.31% of them were central obesity; 30.40% of them felt fatigue/asthenia/drowsiness, 8.85% of them had a self-reported history of diabetes. The demographic characteristics of all participants were shown in Table 1.

**Table 1:** Demographic characteristics of participants.

**Characteristics**	**Number**	**Percentage (%)**
**Gender**	
Male	41,152	41.23
Female	58,669	58.77
**Age (years)**	
35–44	10,042	10.06
45–54	31,183	31.24
55–64	27,466	27.52
65–75	31,130	31.19
**Educational level**	
Illiterate/semi-literate	47,712	47.80
Primary school	21,566	21.60
Junior high school and above	30,543	30.60
**Profession**	
Non-farmer	33,753	33.81
Farmer	66,068	66.19
**Marital status**	
Married	91,577	91.74
Unmarried	8,244	8.26
**Annual household income (yuan)**	
<10,000	18,176	18.21
10,001 25,000	32,696	32.75
>25,000	40,197	40.27
Not sure/refused to answer	8,752	8.77
**Urban or rural**	
Urban	29,389	29.44
Rural	70,432	70.56
**Region**	
Northern Anhui	21,902	21.94
Central Anhui	31,631	31.69
Southern Anhui	46,288	46.37
**Smoking**	
No	79,320	79.46
Yes	20,501	20.54
**Drinking**	
No	83,065	83.21
Yes	16,756	16.79
**BMI(NHFPC)**	
Normal	43,691	43.77
Low body weight	2,523	2.53
Overweight	38,804	38.87
Obesity	14,803	14.83
**BMI (International standard)**	
Normal	56,091	56.19
Low body weight	2,523	2.53
Overweight	34,862	34.92
Obesity	6,284	6.30
**Waistline (NHFPC)**	
Normal	39,870	39.94
Central obesity prophase	21,708	21.75
Central obesity	38,243	38.31
**Waistline (International standard)**	
Normal	82,609	82.76
Abdominal obesity	17,212	17.24
**Snoring while sleeping**	
No	40,157	40.23
Yes	50,923	51.01
Not sure	8,741	8.76
**Fatigue and tiredness**	
No	64,073	64.19
Yes	30,346	30.40
Not sure	5,402	5.41
**History of diabetes**	
No	90,982	91.15
Yes	8,839	8.85
Total	99,821	100.00

### Rate and classification of high-risk groups of CVD

Among the 99,821 participants, the detection rate of high-risk groups was 21.46%. Among high-risk groups, the proportions of CVD history type, hypertension type, dyslipidemia type and high-risk type were 20.33%, 67.37%, 18.94%, and 21.74%, respectively (Table 2). According to the high-risk types, the high-risk groups can be clustered. 74.57% of them had only one high-risk type, 22.57% of them had two high-risk types, and 2.86% had three or more high-risk types.

**Table 2:** Classification of high-risk groups for CVD.

**Type**	**Number**	**Percentage (%)**
CVD history type	4,356	20.33
Hypertension type	14,435	67.37
Dyslipidemia type	4,058	18.94
High risk type	4,658	21.74

### Detection rate of high-risk groups of CVD among residents with different characteristics

Regarding the detection rate of high-risk groups of CVD, men was higher than women, farmers were higher than other occupational groups, rural areas were higher than urban areas, smokers were higher than non-smokers, drinkers were higher than non-drinkers, and married residents was lower than those of other marital status (all *P* < 0.05). There were significant differences in detection rates among residents with different BMI, waist circumference, region, different snoring status, and different fatigue and sleepiness status (all *P* < 0.05). The detection rate of high-risk groups increased with age, and decreased with educational level and annual family income (all *P* < 0.05). The detection rates of high-risk groups are listed in Table 3.

**Table 3:** Comparison of the detection of high-risk groups of CVD among residents with different characteristics.

**Characteristics**	**Number of high-risk groups**	**Detection rate (%)**	**χ^2^ value**	***P* value**
**Gender**			45.98	<0.001
Male	9,266	22.52	
Female	12,160	20.73	
**Age (years)**			3,206.12	<0.001
35–44	941	9.37	
45–54	4,698	15.07	
55–64	6,236	22.70	
65–75	9,551	30.68	
**Educational level**			480.53	<0.001
Illiterate/semi-literate	11,557	24.22	
Primary school	4,467	20.71	
Junior high school and above	5,402	17.69	
**Profession**			71.22	<0.001
Non-farmer	6,727	19.93	
Farmer	14,699	22.25	
**Marital status**			245.51	<0.001
Married	19,097	20.85	
Unmarried	2,329	28.25	
**Annual household income (yuan)**			425.44	<0.001
<10,000	4,690	25.8	
10,001~25,000	7,450	22.79	
>25,000	7,527	18.73	
Not sure/refused to answer	1,759	20.1	
**Urban or rural**			13.25	<0.001
Urban	6,093	20.73	
Rural	15,333	21.77	
**Region**			23.71	<0.001
Northern Anhui	4,484	20.47	
Central Anhui	6,725	21.26	
Southern Anhui	10,217	22.07	
**Smoking**			6.40	0.011
No	16,893	21.3	
Yes	4,533	22.11	
**Drinking**			113.03	<0.001
No	17,314	20.84		
Yes	4,112	24.54		
**BMI**			1,022.04	<0.001
Normal	7,665	17.54	
Low body weight	334	13.24	
Overweight	9,050	23.32	
Obesity	4,377	29.57	
**Waistline**			1,177.05	<0.001
Normal	6,612	16.58	
Central obesity prophase	4,615	21.26	
Central obesity	10,199	26.67	
**Snoring while sleeping**			765.50	<0.001
No	7,043	17.54	
Yes	12,712	24.96	
Not sure	1,671	19.12	
**Fatigue and tiredness**			370.21	<0.001
No	12,561	19.6	
Yes	7,573	24.96	
Not sure	1,292	23.92	
**History of diabetes**			799.12	<0.001
No	18,487	20.32	
Yes	2,939	33.25	
Total	21,426	21.46	

### The interaction analysis

Given that gender may interact with other factors, the interaction between gender and other influencing factors was analyzed and interactions between gender and marital status, educational level, profession, drinking, snoring and history of diabetes were observed (Supplementary Table 1). Considering the correlation between BMI and waistline, we have analyzed the interaction of BMI and waistline as well. However, no interaction between BMI and waistline was found in the result (Supplementary Table 1).

### The stratified analysis by gender

Due to the interaction between gender and some factors, GLMM analysis was conducted separately in male and female (Tables 4, 5). The result in male indicated that age ≥ 45 years old, not married, obtained primary school educational level, annual family income > 25,000 yuan, drinking, low body weight, overweight and obesity, pre-central obesity and central obesity, snoring, feeling fatigued, sleepiness, and self-reported history of diabetes are statistically related to the risk of CVD (Table 4). While in female, age ≥ 45 years old, obtained junior high school and above educational level, occupation as a farmer, annual family income >25,000 yuan, low body weight, overweight and obesity, pre-central obesity and central obesity, snoring, feeling fatigued, sleepiness, and self-reported history of diabetes may be influencing factors of CVD (Table 5).

**Table 4:** Generalized Linear Mixed Model analysis of influencing factors among high-risk groups of CVD in male.

**Characteristics**	**Generalized Linear Mixed Model analysis**		
	**β**	***Z* value**	***P* value**
**Age (years)**	
35–44	
45–54	0.215	3.95	<0.001
55–64	0.580	10.70	<0.001
65–75	0.981	18.13	<0.001
**Marital status**	
Married	
Unmarried	−0.269	−5.98	<0.001
**Educational level**	
Illiterate/semi-literate	
Primary school	0.074	2.28	0.023
Junior high school and above	0.013	0.39	0.697
**Profession**	
Non-farmer	
Farmer	0.023	0.75	0.451
**Annual household income (yuan)**	
<10,000	
10,001~25,000	−0.025	−0.70	0.486
>25,000	−0.083	−2.16	0.031
Not sure/refused to answer	−0.135	−2.56	0.010
**Smoking**	
No	
Yes	−0.028	−1.13	0.259
**Drinking**	
No	
Yes	0.175	6.79	<0.001
**BMI**	
**Normal**	
Low body weight	−0.304	−3.40	<0.001
Overweight	0.232	7.06	<0.001
Obesity	0.524	11.21	<0.001
**Waistline**	
Normal	
Central obesity prophase	0.175	4.83	<0.001
Central obesity	0.308	8.22	<0.001
**Snoring while sleeping**	
No	
Yes	−0.178	−6.35	<0.001
Not sure	−0.129	−2.66	0.008
**Fatigue and tiredness**	
No	
Yes	−0.155	−5.73	<0.001
Not sure	−0.022	−0.40	0.693
**History of diabetes**	
No	
Yes	0.514	13.13	<0.001

**Table 5:** Generalized Linear Mixed Model analysis of influencing factors among high-risk groups of CVD in female.

**Characteristics**	**Generalized linear mixed model analysis**		
	**β**	***Z* value**	***P* value**
**Age (years)**	
35–44	
45–54	0.629	11.39	<0.001
55–64	1.151	20.70	<0.001
65–75	1.578	27.96	<0.001
**Marital status**	
Married	
Unmarried	−0.059	−1.68	0.092
**Educational level**	
Illiterate/semi-literate	
Primary school	−0.030	−1.01	0.314
**Junior high school and above**	−0.096	−2.80	0.005
Profession	
Non-farmer	
Farmer	0.156	5.85	<0.001
**Annual household income (yuan)**	
<10000	
10001~25000	0.014	0.48	0.632
>25000	−0.141	−4.47	<0.001
Not sure/refused to answer	−0.100	−2.33	0.020
**Smoking**	
No	
Yes	−0.050	−0.64	0.523
**Drinking**	
No	
Yes	−0.008	−0.15	0.885
**BMI**	
Normal	
Low body weight	−0.526	−6.22	<0.001
Overweight	0.226	8.03	<0.001
Obesity	0.458	12.27	<0.001
**Waistline**	
Normal	
Central obesity prophase	0.173	5.48	<0.001
Central obesity	0.199	6.02	<0.001
**Snoring while sleeping**	
No	
Yes	−0.258	−10.91	<0.001
Not sure	−0.297	−7.58	<0.001
**Fatigue and tiredness**	
No	
Yes	−0.208	−9.04	<0.001
Not sure	−0.076	1.62	0.105
**History of diabetes**	
No			
Yes	0.354	10.85	<0.001

## Discussion

The impact of location and environmental conditions, and the differences in socioeconomic development, geographical environment, dietary habits, health resources, and health services among different provinces in China lead to a complex geographic distribution of CVD risk in China. The risk levels of the population in each region are significantly different (9). Anhui Province has a relatively high CVD mortality rate among provinces in China (17, 18). In our study, the detection rate of high-risk groups was 21.46% among 8 counties (districts) of Anhui Province.

It was found that the detection rate of high-risk groups of CVD among male and female was significant different in this survey, which has been suggested to be related to higher tobacco consumption, higher drinking frequency and alcohol consumption in men (19, 20). The result of this study also confirmed that drinking was the influencing factor of CVD in male while no significance in female. However, in this study we did not observe any interaction between gender and smoking, which may be because that the data of smoking we used was qualitative and did not perform quantitative analysis on smoking. It has been reported that heavy drinking has a dose-dependent effect on the increasing of blood pressure, and can increase the risk of diabetes, which all contribute to the increased risk of CVD (20–22). Excessive drinking can also trigger ventricular arrhythmias and sudden cardiac arrest by prolongating QT interval and shortening the atrial effective refractory period (23). Similar evidences were also observed in this study, that the most common type of high-risk groups of CVD was the high blood pressure type (67.37%), suggesting that high blood pressure may be a primary risk factor for CVD. Ettehad D et al. (24) have pointed out that every 10 mmHg decrease in systolic blood pressure is associated with a reduced risk of CVD events, and hypertension is associated with a continuous and graded risk of CVD (25). These all suggest us that through early graded intervention, improving the behavioral habits of high-risk groups and controlling the blood pressure level of hypertensive people, the incidence of CVD may be reduced.

In this study, stratified analysis showed that with primary school level in male and with junior high school or above educational level in female may be protective factors for CVD, which was consistent with the results of previous studies (26, 27). This may be attribute to the fact that people with higher education levels tend to pay more attention to their health and willing to adopt a healthy lifestyle to reduce the risk of CVD. The study found that farmers are more likely to be a high-risk group of CVD than non-farmers in female, but not in male. It has also been indicated that women who worked in agriculture trend to have a higher risk of cerebral infarction, while man did not (28). The specific reason for the difference between male and female needed to be further explored. According to GLMM analysis, it was also found that people with lower annual family income are more likely to become a high-risk group of CVD, suggesting a relationship between the level of income and CVD. This result was similar to a previous result in China (29) and was explained that may be related to the imbalanced distribution of educational and medical resources in their region (30). The univariate analysis showed that the detection rate of high-risk groups of CVD among rural population were higher compared with urban population, which is consistent with the results of previous study (31). The reasons may be related to the rapid urbanization of rural areas in recent years, economic development, changes in consumption levels and lifestyles, lower education levels and the level of medical services in the region. These results suggest that we should strengthen the intervention on the controllable risk factors of CVD in rural populations, especially farmers. Above evidences indicated that targeted measures should be taken to prevent CVD according to the distribution of medical and educational resources in different regions, and more medical resources should be devoted to individuals living in rural areas and with low education levels.

This survey found that sleep snoring is a risk factor for high-risk groups of CVD. Previous study also found that the reduction of sleep time and sleep quality caused by sleep snoring can lead to an increased risk of CVD, possibly by accelerating the metabolism of glucose and lipids, accelerating obesity, causing metabolic disorders, and triggering type 2 diabetes (32). In this survey, it was also found that the detection rate of high-risk groups of CVD is elevated accompanied with the increase of BMI and waistline. It might be result from that individual with higher BMI have higher circulating blood volume, which in turn increases cardiac output, leading to increased burden on the heart, and resulting in a series of CVD events (33, 34). As we all know, obesity is one of the most important public health problems with increasing trend worldwide (35). Obesity is not only an independent risk factor for CVD, but also closely associated with several other risk factors, such as hypertension and diabetes. People with a self-report history of diabetes were observed to be at a higher risk of CVD than those without the history in this survey. Studies reported that diabetes increases the risk of CVD three to four–fold in female and two to three–fold in male (36) and CVD is also the main death reason in diabetic patients (37). In conclusion, the prevention of obesity and diabetes is a key link in the prevention of cardiovascular disease. Although population-wide strategies have been adopted to educate people of all ages, especially children, pregnant women and the elderly (38), with the aging of the population, changes in lifestyle, reduction in physical activity and changes in dietary habits, leading to the increasing incidence of obesity and the prevalence of diabetes year by year (39). Therefore, for high-risk groups, it is necessary to actively take primary and secondary preventive measures to prevent obesity and diabetes, so as to further reduce the incidence of cardiovascular disease, and provide targeted dietary guidance and behavioral interventions.

There are some limitations in the study. Firstly, a convenience sampling approach was employed to recruit amounts of participants in this study, so the representativity and generalizability of our study for the whole Anhui province are limited. Secondly, as a kind of observational study, the ability of confirming the causal relationship between these influencing factors and CVD is restricted. We make up for these limitations through the following aspects. Firstly, the sample of our study is considerable huge to reflect the detection rate and influencing factors of high-risk groups of CVD in Anhui to some extent. Second, the interaction between multiple influencing factors was analyzed to reduce the influence of confounding factors on the results. In addition, GLMM analysis was conducted separately in different gender populations.

## Conclusions

The results of this study suggest that the detection rate of high-risk groups of cardiovascular disease in Anhui Province is relatively high, the high risk-groups of CVD in different regions of Anhui Province have different epidemiological characteristics. Therefore, in the future, we should combine the high-risk group strategy with the whole population strategy and take targeted preventive strategies and measures. We should strengthen publicity and education to promote healthy lifestyles, such as no smoking or drinking, strengthening exercise, preventing obesity, etc. We also can identify high-risk groups through early screening in areas with high incidence of CVD, especially those who are already at high risk of hypertension or diabetes.

## Data availability statement

The raw data supporting the conclusions of this article will be made available by the authors, without undue reservation.

## Ethics statement

The studies involving human participants were reviewed and approved by the Ethics Committee of the Anhui Medical University. The patients/participants provided their written informed consent to participate in this study.

## Author contributions

S-ST and Z-RL guided on the design and statistical analyses. X-YX, Z-XW, and Y-WC wrote the manuscript. X-YX had primary responsibility for final content. X-YW, LZ, Y-JC, H-DW, J-QX, and M-XN contributed to data collection and interpretation of findings. All authors contributed to the manuscript editing, read, and approved the final manuscript.

## Conflict of interest

The authors declare that the research was conducted in the absence of any commercial or financial relationships that could be construed as a potential conflict of interest.

## Publisher's note

All claims expressed in this article are solely those of the authors and do not necessarily represent those of their affiliated organizations, or those of the publisher, the editors and the reviewers. Any product that may be evaluated in this article, or claim that may be made by its manufacturer, is not guaranteed or endorsed by the publisher.

## References

[1] Kahleova H Levin S Barnard ND. Vegetarian dietary patterns and cardiovascular disease. Prog Cardiovasc Dis. (2018) 61:54–61. 10.1016/j.pcad.2018.05.002 29800598

[2] Du A Patel SA. D C, Jda E, Cm A. Epidemiology of cardiovascular disease in China and opportunities for improvement. J Am Coll Cardiol. (2019) 73:3135–47. 10.1016/j.jacc.2019.04.036 31221263

[3] Chen WW Gao RL Liu LS Zhu ML Wang W Wang YJ. Report on cardiovascular disease in China (2013). Zhongguo Xun Huan Za Zhi. (2015) 29:487–91.

[4] Collaborators GMaCoD. Global, regional, and national age-sex specific all-cause and cause-specific mortality for 240 causes of death, 1990-2013: a systematic analysis for the global burden of disease study 2013. Lancet. (2015) 385:117–71. 10.1016/S0140-6736(14)61682-2 25530442 PMC4340604

[5] He L Tang X Song Y Li N Li J Zhang Z. Prevalence of cardiovascular disease and risk factors in a rural district of Beijing, China: a population-based survey of 58,308 residents. BMC Public Health. (2012) 12:34. 10.1186/1471-2458-12-34 22248490 PMC3292979

[6] Bundy JD He J. Hypertension and related cardiovascular disease burden in China. Ann Glob Health. (2018) 82:227–33. 10.1016/j.aogh.2016.02.002 27372527

[7] World Health Organization. Prevention of Recurrent Heart Attacks and Strokes in Low-and Middle-Income Populations: Evidence-Based Recommendations for Policy-Makers and Health Profissionals. Geneva: Publications of the World Health Organization. (2003)

[8] Qin H Chen Y Da ND Wei X Liu Z. Analysis on death causes of residents in Anhui province, 2013. Zhonghua Liu Xing Bing Xue Za Zhi. (2015) 36:976. 10.3760/cma.j.issn.0254-6450.2015.09.015 26814866

[9] Zhao D Liu J Wang M Zhang X Zhou M. Epidemiology of cardiovascular disease in China: current features and implications. Nat Rev Cardiol. (2019) 16:203–12. 10.1038/s41569-018-0119-4 30467329

[10] Lu J Xuan S Downing NS Wu C Li L Krumholz HM. Protocol for the China PEACE (patient-centered evaluative assessment of cardiac events) million persons project pilot. BMJ Open. (2016) 6:e010200. 10.1136/bmjopen-2015-010200 26729395 PMC4716208

[11] Ghorpade AG Shrivastava SR Kar SS Sarkar S Majgi SM Roy G. Estimation of the cardiovascular risk using world health organization/international society of hypertension (WHO/ISH) risk prediction charts in a rural population of South India. Int J Health Policy Manag. (2015) 4:531–6. 10.15171/ijhpm.2015.88 26340393 PMC4529043

[12] Li X Wu C Lu J Chen B Li Y Yang Y. Cardiovascular risk factors in China: a nationwide population-based cohort study. Lancet Public Health. (2020) 5:e672–81. 10.1016/S2468-2667(20)30191-2 33271080

[13] World Health Organization cardiovascular disease risk charts: revised models to estimate risk in 21 global regions. Lancet Glob Health. (2019) 7:e1332–45. 10.1016/S2214-109X(19)30318-3 31488387 PMC7025029

[14] WorldHealthOrganization. Prevention of Cardiovascular Disease. Guidelines for Assessment and Management of Cardiovascular risk. Geneva: WHO Press, World Health Organization (2007).

[15] BeiFan Z. China CM-AGotWGoOi. Predictive values of body mass index and waist circumference for risk factors of certain related diseases in Chinese adults–study on optimal cut-off points of body mass index and waist circumference in Chinese adults. Biomed Environ Sci. (2002) 15:83–96. 10.1046/j.1440-6047.11.s8.9.x 12046553

[16] Ross R Neeland IJ Yamashita S Shai I Seidell J Magni P. Waist circumference as a vital sign in clinical practice: a consensus statement from the IAS and ICCR working group on visceral obesity. Nat Rev Endocrinol. (2020) 16:177–89. 10.1038/s41574-019-0310-7 32020062 PMC7027970

[17] Zhou M Wang H Zhu J Chen W Wang L Liu S. Cause-specific mortality for 240 causes in China during 1990-2013: a systematic subnational analysis for the global burden of disease study 2013. Lancet. (2016) 387:251–72. 10.1016/S0140-6736(15)00551-6 26510778

[18] Liu S Li Y Zeng X Wang H Yin P Wang L. Burden of cardiovascular diseases in China, 1990-2016: findings from the 2016 global burden of disease study. JAMA Cardiol. (2019) 4:342–52. 10.1001/jamacardio.2019.0295 30865215 PMC6484795

[19] Moran AE Forouzanfar MH Roth G Ezzati M Mensah G Flaxman A. PM278 The global burden of ischemic heart disease in 1990 and 2010: the global burden of disease 2010 study. Glob Heart. (2014) 9:e117. 10.1016/j.gheart.2014.03.1641 24573351 PMC4181601

[20] Li Z Bai Y Guo X Zheng L Sun Y Roselle AM. Alcohol consumption and cardiovascular diseases in rural China. Int J Cardiol. (2016) 215:257–62. 10.1016/j.ijcard.2016.04.095 27128542

[21] Ronksley PE Brien SE Turner BJ Mukamal KJ Ghali WA Ronksley PE. Association of alcohol consumption with selected cardiovascular disease outcomes: a systematic review and meta-analysis. BMJ. (2011) 342:d671. 10.1136/bmj.d671 21343207 PMC3043109

[22] Polsky S Akturk HK. Alcohol consumption, diabetes risk, and cardiovascular disease within diabetes. Curr Diab Rep. (2017) 17:136. 10.1007/s11892-017-0950-8 29103170

[23] O'Keefe EL DiNicolantonio JJ O'Keefe JH Lavie CJ. Alcohol and CV health: Jekyll and Hyde J-curves. Prog Cardiovasc Dis. (2018) 61:68–75. 10.1016/j.pcad.2018.02.001 29458056

[24] Ettehad D Emdin CA Kiran A Anderson SG Callender T Emberson J. Blood pressure lowering for prevention of cardiovascular disease and death: a systematic review and meta-analysis. Lancet. (2016) 387:957–67. 10.1016/S0140-6736(15)01225-8 26724178

[25] Vasan RS. High blood pressure in young adulthood and risk of premature cardiovascular disease: calibrating treatment benefits to potential harm. JAMA. (2018) 320:1760–3. 10.1001/jama.2018.16068 30398583

[26] Delpierre C Lauwers-Cances V Datta GD Berkman L Lang T. Impact of social position on the effect of cardiovascular risk factors on self-rated health. Am J Public Health. (2009) 99:1278–84. 10.2105/AJPH.2008.147934 19443823 PMC2696651

[27] Yusuf S Joseph P Rangarajan S Islam S Mente A Hystad P. Modifiable risk factors, cardiovascular disease, and mortality in 155 722 individuals from 21 high-income, middle-income, and low-income countries (PURE): a prospective cohort study. Lancet. (2020) 395:795–808. 10.1016/S0140-6736(19)32008-2 31492503 PMC8006904

[28] Fukai K Furuya Y Nakazawa S Kojimahara N Hoshi K Toyota A. A case control study of occupation and cardiovascular disease risk in Japanese men and women. Sci Rep. (2021) 11:23983. 10.1038/s41598-021-03410-9 34907236 PMC8671491

[29] Yan R Li W Yin L Wang Y Bo J. Cardiovascular diseases and risk-factor burden in urban and rural communities in high-, middle-, and low-income regions of China: a large community-based epidemiological study. J Am Heart Assoc Cardiovascul Cerebrovascular Disease. (2017) 6:e004445. 10.1161/JAHA.116.004445 28167497 PMC5523752

[30] Li W Gu H Teo KK Bo J Wang Y Yang J. Hypertension prevalence, awareness, treatment, and control in 115 rural and urban communities involving 47 000 people from China. J Hypertens. (2016) 34:39–46. 10.1097/HJH.0000000000000745 26630211

[31] Wu J Cheng X Qiu L Xu T Zhu G Han J. Prevalence and clustering of major cardiovascular risk factors in China: a recent cross-sectional survey. Medicine (Baltimore). (2016) 95:e2712. 10.1097/MD.0000000000002712 26962771 PMC4998852

[32] Yu X Li ZB Cjl C Xin QC Yqf A Jiang S. et al. Predicted 10-year cardiovascular disease risk and its association with sleep duration among adults in Beijing-Tianjin-Hebei Region, China. Sci Direct. (2021) 34:803–13. 10.3967/bes2021.109 34782046

[33] Salvadori A Fanari P Mazza P Agosti R Longhini E. Work capacity and cardiopulmonary adaptation of the obese subject during exercise testing. Chest. (1992) 101:674–9. 10.1378/chest.101.3.674 1541131

[34] Lavie CJ Milani RV Ventura HO. Obesity and cardiovascular disease: risk factor, paradox, and impact of weight loss - sciencedirect. J Am Coll Cardiol. (2009) 53:1925–32. 10.1016/j.jacc.2008.12.068 19460605

[35] Ortega FB Lavie CJ Blair SN. Obesity, Diabetes, and Cardiovascular Diseases Compendium. Circ Res. (2016) 118:1703–5. 10.1161/CIRCRESAHA.116.308999 27230636 PMC4888905

[36] Norhammar A Schenck-Gustafsson K. Type 2 diabetes and cardiovascular disease in women. Diabetologia. (2013) 56:1–9. 10.1007/s00125-012-2694-y 22945305

[37] Rao Kondapally Seshasai S Kaptoge S Thompson A Di Angelantonio E Gao P Sarwar N. Diabetes mellitus, fasting glucose, and risk of cause-specific death. N Engl J Med. (2011) 364:829–41. 10.1056/NEJMoa1008862 21366474 PMC4109980

[38] Pearce C Rychetnik L Wutzke S Wilson A. Obesity prevention and the role of hospital and community-based health services: a scoping review. BMC Health Serv Res. (2019) 19:453. 10.1186/s12913-019-4262-3 31277640 PMC6612151

[39] Saeedi P Petersohn I Salpea P Malanda B Karuranga S Unwin N. Global and regional diabetes prevalence estimates for 2019 and projections for 2030 and 2045: results from the international diabetes federation diabetes atlas, 9(th) edition. Diabetes Res Clin Pract. (2019) 157:107843. 10.1016/j.diabres.2019.107843 31518657

