# Supplementary material for: The detection rate and influencing factors of high-risk groups of cardiovascular disease in Anhui, China: A cross-sectional study of 99,821 residents
Source: Front Public Health. 2022 Aug 25;10:921038. doi: 10.3389/fpubh.2022.921038 (PMC9454339; doi:10.3389/fpubh.2022.921038)
Supplement: Supplementary file 1 [file Table_1.docx]

Supplementary Table 1 The interaction analysis of influencing factors among high-risk groups of CVD

| Factors | β | | | *Z* value | *P* value | |
| --- | --- | --- | --- | --- | --- | --- |
| Gender(Female): Marital status (Married) | 0.184 | | | 3.25 | 0.001 | |
| Gender(Female): Educational level (Primary school) | -0.154 | | | -3.56 | <0.001 | |
| Gender(Female): Educational level (Junior high school and above) | -0.207 | | | -4.51 | <0.001 | |
| Gender(Female) Profession (Farmer) | 0.115 | | | 2.89 | 0.004 | |
| Gender(Female): Annual household income (yuan) 10001～25000 | 0.024 | | | 0.52 | 0.603 | |
| Gender(Female): Annual household income (yuan) >25000 | -0.088 | | | -1.78 | 0.076 | |
| Gender(Female): Annual household income (yuan)Not sure/refused to answer | 0.001 | | | 0.01 | 0.991 | |
| Gender(Female): Smoking(Yes) | -0.020 | | | -0.24 | 0.807 | |
| Gender(Female): Drinking(Yes) | -0.165 | | | -2.84 | 0.004 | |
| Gender(Female): BMI(Low body weight) | -0.193 | | | -1.57 | 0.116 | |
| Gender(Female): BMI(Overweight) | -0.025 | | | -0.57 | 0.567 | |
| Gender(Female): BMI(Obesity) | -0.108 | | | -1.82 | 0.069 | |
| Gender(Female): Waistline (Central obesity prophase) | 0.003 | | | 0.07 | 0.945 | |
| Gender(Female): Waistline (Central obesity) | -0.097 | | | -1.95 | 0.051 | |
| Gender(Female): Snoring while sleeping(Yes) | -0.095 | | | -2.62 | 0.009 | |
| Gender(Female): Snoring while sleeping(Not sure) | -0.177 | | | -2.87 | 0.004 | |
| Gender(Female): Fatigue and tiredness(Yes) | -0.065 | | | -1.83 | 0.068 | |
| Gender(Female): Fatigue and tiredness(Not sure) | 0.087 | | | 1.19 | 0.235 | |
| Gender(Female): History of diabetes(Yes) | -0.131 | | | -2.58 | 0.010 | |
| BMI(Low body weight): Waistline(Central obesity prophase) | 0.107 | | | 0.33 | 0.742 | |
| BMI(Overweight): Waistline(Central obesity prophase) | -0.058 | | | -1.17 | 0.241 | |
| BMI(Obesity): Waistline(Central obesity prophase) | | -0.054 | -0.31 | | | 0.757 |
| BMI(Low body weight): Waistline( Central obesity) | | 0.354 | 0.743 | | | 0.457 |
| BMI(Overweight): Waistline(Central obesity) | | -0.051 | -0.96 | | | 0.339 |
| BMI(Obesity): Waistline(Central obesity) | | -0.093 | -0.59 | | | 0.558 |
